# Problem Formulation in Knowledge Discovery via Data Analytics (KDDA) for Environmental Risk Management

**DOI:** 10.3390/ijerph13121245

**Published:** 2016-12-15

**Authors:** Yan Li, Manoj Thomas, Kweku-Muata Osei-Bryson, Jason Levy

**Affiliations:** 1 Center for Information Systems and Technology (CISAT), Claremont Graduate University, 130 E. Ninth St. ACB225, Claremont, CA 91711, USA; yan.li@cgu.edu; 2 Information Systems, Virginia Commonwealth University, Richmond, VA 23284, USA; mthomas@vcu.edu (M.T.); kmosei@vcu.edu (K.-M.O.-B.); 3 Public Administration, University of Hawaii, West Oahu, Kapolei, HI 97607, USA

**Keywords:** Knowledge Discovery via Data Analytics (KDDA), problem formulation, decision support, environmental risk, ontology

## Abstract

With the growing popularity of data analytics and data science in the field of environmental risk management, a formalized Knowledge Discovery via Data Analytics (KDDA) process that incorporates all applicable analytical techniques for a specific environmental risk management problem is essential. In this emerging field, there is limited research dealing with the use of decision support to elicit environmental risk management (ERM) objectives and identify analytical goals from ERM decision makers. In this paper, we address problem formulation in the ERM understanding phase of the KDDA process. We build a DM^3^ ontology to capture ERM objectives and to inference analytical goals and associated analytical techniques. A framework to assist decision making in the problem formulation process is developed. It is shown how the ontology-based knowledge system can provide structured guidance to retrieve relevant knowledge during problem formulation. The importance of not only operationalizing the KDDA approach in a real-world environment but also evaluating the effectiveness of the proposed procedure is emphasized. We demonstrate how ontology inferencing may be used to discover analytical goals and techniques by conceptualizing Hazardous Air Pollutants (HAPs) exposure shifts based on a multilevel analysis of the level of urbanization (and related economic activity) and the degree of Socio-Economic Deprivation (SED) at the local neighborhood level. The HAPs case highlights not only the role of complexity in problem formulation but also the need for integrating data from multiple sources and the importance of employing appropriate KDDA modeling techniques. Challenges and opportunities for KDDA are summarized with an emphasis on environmental risk management and HAPs.

## 1. Introduction

Scholarly interest pertaining to the theory and practice of environmental risk management (ERM) analytics and data science has exploded over the past decade. This broad field refers to the systems, technologies, and methodologies for the continuous improvement and iterative investigation of past ERM results to make future recommendations, find pareto-optimal solutions, and drive future ERM planning. There are different types of analytics that can be applied in the decision support process for ERM. They include descriptive analytics (wherein the analyst gains insight from archival data to shed light on what occurred historically, often using reporting, scorecards, clustering, etc.), diagnostics (a more detailed type of descriptive analytics by drilling down and interacting with data to answer questions about outcomes, events, or trends), predictive analytics (predictive modeling using statistical and machine learning techniques), and prescriptive analytics (wherein analysts normatively recommend decisions using tools for optimization, simulation, etc.). Over the past few years, the application of analytics and data science has become an integral component of environmental risk management decision making. A number of ERM analytics and data science domains have recently emerged. For example, behavioral analytics investigate how (and why) users of ERM platforms and applications behave in a given manner. Other emerging fields include risk and credit analytics, collections analytics, financial services analytics, fraud analytics, marketing/pricing analytics, and supply chain/logistics analytics. Advances in ERM analytics and data science are attributable to several factors, including advances in information technologies such as mobile, analytics, big data, social networking, and cloud computing. These techniques serve as both the drivers and enablers for the adoption and use of analytics in ERM. For the purpose of this paper, the term analytics is defined as “*the analysis of data, using sophisticated quantitative methods, to produce insights that traditional approaches to ERM Intelligence are unlikely to discover*” [1].

The fundamental concepts of data science are drawn from data mining (DM) and data analytics [2]. Data mining is the use of algorithms, methods, and tools used for analyzing data or extracting patterns. It is the process of exploration and analysis of large quantities of data, through computer-based machine learning techniques integrated with statistical algorithms, to discover previous unknown and potentially useful patterns and rules [3]. The term Knowledge Discovery and Data Mining (KDDM) has been proposed for the overall knowledge discovery process using data mining [4]. Data analytics, however, involves a wider range of quantitative methods than traditional machine learning/data mining methods and algorithms. In addition to the traditional data mining algorithms and techniques, such as tree induction, neural networks, clustering analysis, support vector machines, association rules, etc., data analytics also include discrete event simulation, multiple attribute decision analysis (MADA), mathematical optimization, and visualization. All three areas (i.e., KDDM, data analytics, and data science) are very closely related and share concepts related to extracting and creating knowledge from data to solve ERM problems. In this paper, we use the term Knowledge Discovery via Data Analytics (KDDA) to describe the knowledge discovery process and practices. The rationale for this adaptation over the traditional KDDM can be summarized as follows:

1. Each different analytical technique has its own unique requirements based on its fit to the environmental risk management objectives, on its data input and transformation needs, and on its output evaluation and deployment. While the number of analytical techniques continuously grows, a formalized knowledge discovery process that incorporates all applicable analytical techniques for a specific environmental risk management problem is essential. KDDA extends the current KDDM practices and capture such a requirement.
2. KDDA provides a research lens that focuses on problems that are relevant to practitioners and the state-of-art of analytical application development and implementation technologies.

The popularity of data analytics and data science in ERM comes from the clear articulation of problem solving as an end goal. Similar to many other problem domains, a key attribute for successful analytics for ERM is the ability to articulate ill-structured problem into analytical questions that can be answered by KDDA techniques. When describing a KDDA project life cycle or KDDA process, practitioners may adopt traditional KDDM process models in order to translate very technical analytical solutions (such as complex algorithms, matrices, criteria, and so forth) into information that is applicable and relevant to the individual case of ERM. This is especially true for KDDA initiatives that center around problem formulation, including the identification and contextualizing of objectives.

A challenging problem in Information Systems (IS) research involves helping various types of users avoid many common analytical mistakes by improving the automation of some aspects of the knowledge discovery process [5]. In the context of problem formulation in KDDA, there are three major concerns in the organizational setting. First, there is the need to translate very-technical descriptions and solutions into domain (e.g., ERM) specific language, which in turn, will be a significant factor in integrating the KDDA solutions into the decision making process [6]. Current approaches towards the decision support in the KDDA process are mainly from a knowledge engineer’s perspective, resulting in a semantic gap between decision makers (who are interested in applied concepts, such as criteria air pollutants and hazardous air pollutant rate) and knowledge engineers (who focus on technical constructs, such as chemical risk scoring and bias-correlation). In addition, there may be subjective objectives and success criteria that make the translation from ERM terminology to KDDA terminology even more problematic.

Second, there are inherent limitations in the human’s ability to recall. It is well known that there are limitations on human short-term memory that can affect recall of relevant information concerning both organizational and domain knowledge. This fact is important during the problem formulation in the environmental risk management understanding (ERMU) phase of the KDDA process where stakeholders are expected to identify all relevant objectives and define them appropriately. This limitation can also lead to challenges for stakeholders that are “experts” with respect to some dimensions of the relevant decision-making problem. This may lead to some experts being inappropriately impacted by informational influence, the acceptance of evidence from others as evidence of reality.

Third, there is the need to support group decision making. The problem formulation in the KDDA process typically involves multiple stakeholders who may have different values and different opinions with regards to objectives that are relevant, relationships between the objectives, and the relative importance of each objective. There is thus the need for a process to provide decision guidance to empower group members to successfully face the challenge of consensus building.

The rest of this paper is organized as follows. We first provide background on problem formulation in the KDDA process and discuss key methods for assessing and supporting group consensus and knowledge discovery (Section 2). A framework for supporting ERM problem formulation is presented next (Section 3). We then describe the ontology-based approach for identifying analytical techniques and goals in Section 4, followed by a case study of applying our proposed framework in an environmental epidemiology use case (Section 5). We demonstrate how ontology inferencing may be used to discover analytical goals and techniques by conceptualizing Hazardous Air Pollutants (HAPs) exposure hazard shifts based on a multilevel analysis of the level of urbanization and related economic activity and the degree of socioeconomic deprivation (SED) at the local neighborhood level [7]. We conclude with an overview for future research directions and barriers and bridges to innovations in KDDA in Section 6. 

## 2. Problem Formulation in the KDDA Process

In order to use KDDA to solve ERM problems more efficiently, a process model is desired to support the integration of KDDA solutions into ERM processes. Many knowledge discovery process models have been developed in academia and in industry to help organizations understand the knowledge discovery processes, and to organize the knowledge discovery projects within a common framework [8]. A side-by-side comparison of five major knowledge discovery process models [9] reveals several common features, although each process model has different number of steps and different terminologies for each step. For example, the sequence of steps followed in most of the process models is similar, and the processes are iterative in nature.

The CRISP-DM (CRoss Industry Standard Process for Data Mining) is a robust, well-proven, valuable and popular knowledge discovery process models for use in data mining projects. It was first proposed in 2000 as an industry tool and application-neutral standard process model [10]. The CRISP-DM model is discussed by Shearer [11]. It organizes the DM process into six interdependent phases, namely business understanding, data understanding, data preparation, modeling, evaluation, and deployment.

Business understanding (BU) is considered as the most important phase of any analytical project, and has been highlighted across all existing knowledge discovery process models. When applied in the ERM, the ERMU focuses on understanding ERM objectives and requirements of a data analytics initiative, and then converting this understanding into a defined analytical problem. A large body of scholarship exists pertaining to the provision of decision support for the knowledge discovery process [12,13,14,15] and industry solutions (e.g., Weka, KNIME5, Rapid Miner, SAS Enterprise Miner, SPSS Clementine, etc.). A review of literature reveals that these approaches are largely data-centric and modeling technique-centric. Currently, decision support for BU is very limited.

The second phase in the CRISP-DM model involves initial data collection and understanding the data which involves loading data into a tool. When a complex project involves multiple data sources, it is important to analyze the procedure for integrating all sources of data. Key steps in this phase include data examination, verifying data quality (examining if data is missing or contains errors, etc.) and developing a data quality report. The third phase (data preparation) involves transforming data, improving data and selection (table selection, attribute selection, etc.). All of these steps may be required to produce the final dataset (based on the input data) that will then be used by the fourth phase: modeling. In the modeling phase, applicable modeling techniques are selected, along with a test design for models’ quality and validity, followed by model building and assessment.

There is dynamic interplay between the data preparation phase and modeling phase, particularly when unusual or new data requirements are identified in which case decision makers must return to the data preparation phase. In the modeling phase, optimal parameter values are calculated. Group decision and negotiation support is particularly critical in the fifth and penultimate phase (the step prior to full system deployment): model evaluation and construction. Here, key stakeholders should critically examine the ERM goals and reach a consensus on the optimal KDDA tools. The sixth phase, deployment phase, varies greatly according to the industry and type of problem. Sophisticated deployment involves applying learned knowledge in models within the decision-making process.

KDDA centers around cleared defined business objectives. Clear articulation of ERM strategies and objectives are critical to the success of any analytical project [16]. The performance of analytical programs in an organization has to be measured by how well they help ERM initiatives achieve their strategic objectives. The need to formally capture the ERM objectives and translate them into ERM criteria is not. In this paper, we focus on the problem formulation in the BU phase of the KDDA process.

There is a limited literature on how to provide decision support to ERM objectives and define ERM success criteria based on the environmental risk management requirements for the KDDA process. In the KDDA process, the organization rarely starts with a clearly defined objective. The ERM users are often overwhelmed by the amount of data and hence, require machine capabilities to discover problems that humans cannot comprehend. However, structured or semi-structured decision support can be used by incorporating a variety of goal-elicitation techniques, such as influence diagrams, value-focused thinking (VFT) [17] and Goal Question Metrics (GQM). The ability to capture a KDDA-related ERM goal in a structured or semi-structured way can facilitate the translation [18] of ERM objectives to analytical goals. If possible, ERM objectives can be stored and reused for knowledge management purposes.

A problem can be best defined as an undesirable situation that is expected to be altered or completed in a desired manner, while it is believed to be solvable with some difficulty [19]. “The formulation of a problem is often more essential than its solution...” [20]. Problem formulation has been well recognized as the most important aspect of the decision process [21,22]. However, at a conceptual level, it is different from the traditional concept of “decision making” that involves making a choice of identified alternatives. Problem solving focuses on resolving “the difference between some existing situation and some desired situation” [23]. Thus, the two concepts, “problem solving” and “decision making”, are similar at a cognitive process level, but denote different bodies of research into human thought [24].

The quality of a well-formulated ERM problem can potentially affect the results of succeeding phases in the KDDA process. While previous research mainly focuses on describing and solving well-defined analytical problems, ERM problems in the area of analytics are often ill structured and complex. Literature discerns four types of problem formulation processes as it relates to the clarity of the goal state, based on characteristics of the problem space, based on the set of problem-relevant knowledge, and reference to the problem solving process. Inadequately defined goals can cause problems in validating whether the proposed solution is acceptable. A problem space is a formal, explicit representation of the problem. A well-structured problem [25] shall include a problem space that includes initial state, goal state, and all possible intermediate states; represents all attainable state changes or transformations; shall represents all relevant knowledge; and is isomorphic to the problem involving real-world actions. The assumption of a knowledge-based problem formulation is that the problem solver lacks knowledge in determining the problem structure, relevant states and transformation.

Smith [26] provides a problem taxonomy of problem categories and problem types that can be used as a means to decompose complex problems into sub-problems that match the specific problem solving solution techniques. He proposed four general problem categories: state change (the need to change some unsatisfactory state or to achieve some goal), performance (the need to improve performance of some function or system), knowledge (the need to acquire certain knowledge), and implementation (the need to put some action into effect). Within these categories, the problem type for the KDDA process is related to the knowledge category. Relevant problem types related to the KDDA process are: description (determining what happens to be the case), evaluation (assessing the worth of entity against one’s preferences or external standards), diagnosis (providing explanations of why things are what they are), prediction (predicting future or unknown current states of affairs) and design (determining what one should do to achieve a desired state).

Sharma and Osei-Bryson [26] suggest a four-step guideline towards formulating objectives: (1) apply VFT to stimulate discussion about objectives; (2) apply the GQM approach to generate preliminary statement of objectives; (3) assess preliminary statement of objectives against SMART [27] criteria; and (4) refine the preliminary statement from (2) based on output from (3). The proposed steps provide a structured approach towards formulating ERM objectives. However, it does not fit well in the ill-structured decision context of an analytical environment. Nevertheless, the GQM approach can be adopted to establish measurable goals. The SMART (Specific, Measurable, Achievable, Relevant, and Time-bounded) criteria can be used to assess the ERM objectives.

The ERM problem formulation approaches described above only apply to the individual decision maker. The problem formulation in the BU phase of the KDDA process typically involves multiple stakeholders who may have a plurality of points of views, many of which may be conflicting and need to be handled at the same time. There is thus the need for a process to provide decision guidance to empower group members in consensus building. Group decision and negotiation support plays a valuable role in the knowledge discovery process. The explosion and complexity of data is affecting disciplines from transportation and engineering to government and molecular biology. The latest innovations in knowledge discovery and post-modern information systems are necessary to improve group decision and negotiation support in the “Big data era”. In particular, mobile, pervasive and soft computing are of central importance to dealing with complex and urgent industrial, environmental and social problems associated with a significant increase data velocity, volume, value in the post-industrial age. Group decision support is in ERM, where there is an urgent need to improve human analysis capabilities so that government agencies and corporations are able to manage large volumes of socio-economic and technical information.

KDDA can help to address key challenges relating to group decision making and complex ERM decision making including data overload. Kirker et al. [28] notes that “Decision making in environmental projects can be complex and seemingly intractable, principally because of the inherent trade-offs between sociopolitical, environmental, ecological, and economic factors”. They highlighted the importance of group decision making in ERM, where environmental issues tend to involve shared resources and broad constituencies. However, a number of important issues pertinent to group decision making shall be considered, such as how groups of decision makers in ERM can succeed in making holistic, accurate and timely decisions without the susceptibility of group thinking and entrenched positions. 

Group decision and negotiation support is extremely valuable in the highly competitive KDDA process. As noted in Levy and Taji [29] the Group Analytic Hierarchy Process (GAHP) is a popular tool for modeling the “group prioritization” process that allows decision makers to form a group response for a complex decision problem [30]. Levy and Taji [29] put forth a quadratic programming approach to group support and summarize various issues as it relates to (1) estimating the weights of elements in GAHP; (2) averaging processes for synthesizing reciprocal judgments and (3) social choice axioms pertaining to group preference aggregation [31,32] and (4) pareto optimality [33]. Bryson [34] proposed a framework to assess group consensus and support the group consensus building using a consensus-based GAHP approach [30]. The group consensus decision is presented by a preference vector *W*^GM^ = (*W*_1_,..., *W*_N_), where for (*i*,*j*) in (1,..., N), the ratio *W*_i_/*W*_j_ reflects the group’s belief in the relative importance of element *i* and element *j*. As opposed to the traditional approach to reach a consensus that is simply mathematically derived, the framework derives *W*^GM^ from human interaction by the use of consensus relevant information embedded in the preference data. 

## 3. A Framework for ERM Problem Formulation

In this section, we present a framework for ERM problem formulation. The decision support framework includes four steps, which are problem background description, domain understanding, model ERM objectives, and the identification of analytical techniques and goals. In the following sections, we describe these steps in detail.

### 3.1. Problem Background Description

**Figure 1:**
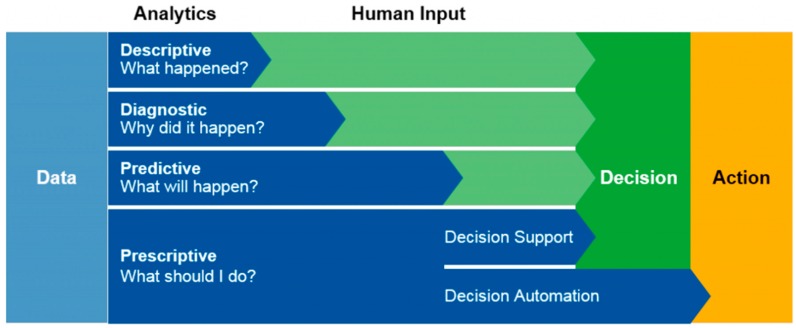
Gartner analytics capabilities framework [34].

Tasks in this step involve obtaining and reviewing organizational mission and vision statements, organizational charts, reviewing any existing organizational ontology, and evaluating the products and services offered. Internal and external stakeholders who are involved in the decision making process are identified. Another key task in this step is to assess the organization’s analytical capability maturity. Specifically, three analytical capability maturity needs to be assessed. They include data maturity (determine suitability of data for analytics), analytic maturity (evaluate the analytical environment of the organization), and decision style maturity (assess the users’ decision styles to use the analytical result). One approach to assess the organizational analytical maturity is to use the Gartner analytics capabilities framework [34] shown in Figure 1.

There are different types of enterprise knowledge, which resides in multiple sources. Multiple perspectives need to be considered in the knowledge acquisition process to avoid potential oversight. Examples of perspectives that include knowledge (i.e., data and information) are, objects that are stored and manipulated, state of knowing and understanding, process of applying expertise, condition of information access, and capability to influence action [35]. Distinction between tacit–explicit and individual–collective knowledge also needs to be considered [36,37]. Explicit knowledge is knowledge that is articulated, codified, and communicated [35]. Tacit knowledge refers to an individual’s cognitive and technical knowledge, and is rooted in the individual's action, experience, and involvement in a specific context [36]. The background tacit knowledge is required for the researcher to acquire and interpret explicit knowledge. In order to acquire tacit knowledge and capture explicit knowledge, a shared knowledge base that is human and machine interpretable will be beneficial.

Chen [38] proposed a conceptual ontology based approach for organizational knowledge representation and reasoning. Even though knowledge within the organization can be difficult and costly to transfer, hard to replicate, and often invisible to outside observers, Chen [38] utilizes the ontology based system to describe the elements, traits, characteristics, and features of the empirical knowledge within the organization. Using a multi-layer approach, the conceptual model divides organizational knowledge into four layers, “know-what”, “know-why”, “know-how”, and “know-with”, to identify the class, hierarchy, layer and composition of empirical knowledge. Reasoning rules are then written using Ontology Web Language—Descriptive Logic (OWL DL) to facilitate the sharing of tacit knowledge. 

### 3.2. Domain Understanding

Domain understanding and its management are widely recognized as critical factors for organizational success and competitive advantage [39]. An organizational ontology similar to Chen [38] provides the set of terms and constraints that describe the structure and behavior of the organization. Noy and McGuinness (2001) highlight several benefits of developing an ontology to make domain assumptions explicit. An OWL based ontology can formalize a domain by defining the relevant concepts of the given decision problem, objectives for the given decision problem, best practices for the given decision problem, and concerns from the technical, technological, legal, learning and innovation perspectives. The ontology can facilitate the sharing of the structure of information among stakeholders in the domain, and assist new entrants to quickly assimilate the domain concepts and knowledge [40].

### 3.3. Model ERM Objectives

The VFT methodology [17] provides guidance on the formulation of objectives. Within the context of the VFT methodology, objectives are classified as either a fundamental objective (FO) or a means objective (MO), where each MO is an objective that is required in order to directly achieve its parent FO or another MO. Although VFT can be conducted in a top-down or bottom-up manner, our focus here is on the former. In the top-down approach, MOs are obtained from the FO by determining all immediate lower level objectives that must be satisfactory in order to achieve the given FO. Lower level MOs can be obtained for the next higher level MOs in a similar manner. The result is a network of objectives with the FOs at the root level and a set of MOs are the leaf level. Each leaf level MO can be considered equivalent to an actionable goal.

The GQM method [41] is a formal approach for generating appropriate measures for a given set of goals. It has been applied in various application areas [18,42,43]. GQM involves the development of a top-down hierarchical structure consisting of three components: goals, questions and metrics. A goal can be refined into a set of questions each of which can be further refined into a set of quantitative and/or qualitative metrics. Our use of the GQM method begins with a set of actionable goals that are leaf level MOs.

Framing the decision situation will thus entail defining the decision context, identifying the objectives, structuring the objectives into a means–ends network, specifying attributes, eliciting preferences of the stakeholders, identifying alternatives, and finally recommending solutions. Give the results from the previous step, we assume that each decision maker would generate an initial set of objectives and initial means–ends network. In the following section, we present a procedure to support consensus assessment and group decision making for a group preference modeling problem. Assisted by a facilitator, group members engage in analysis, discourse and negotiation using the available consensus relevant information including information resulting from the business understanding and domain understanding activities.

#### Procedure for Generating Group Means–Ends Network

**Step 1: Preparation**

- Specify MAXCYCLE, the maximum number of cycles of the group preference elicitation process.
- Specify threshold values for consensus indicators.
- Set CYCLE = 0.

**Step 2: Initial Group Discussion**

- Group members would individually use the ontology to undertake problem background description and domain understanding activities as described in Section 5.1 and Section 5.2.
- The group meets to discuss the given problem situation, and group members offer their opinions along with supporting arguments for different points of view.
- Each group member offers an initial list of FOs and associated children MOs. This involves specifying the intended meaning of each FO in terms of more specific MOs. The member then uses GQM to generate measures.
- The facilitator assists the group in generating a common set of terms for each FO and MO that has been identified at this point.

**Step 3: Determination of Individual Means–Ends Network**

- Set CYCLE = CYCLE + 1.
- Given the list of FOs and MOs identified in the Step 2, each group member further subdivides the objectives until the lowest level is sufficiently well defined that a measurable attribute can be associated with it.
- Each group member generates *Individual Means–Ends Network* MEW^t^.

**Step 4: Computation of Consensus Indicators**

- A *Group Means–Ends Network*, MEW^GM^, is generated from the *Individual Means–Ends Networks*.
- The *Group* and *Individual Consensus Indicators* are calculated.
- Each group member is provided with the *Individual Consensus Indicators*, the *Group Consensus Indicators*, the *Group Means–Ends Network* MEW^GM^, and the similarity of MEW^t^ to MEW^GM^.

**Step 5: Termination Test**

- If the group consensus indicators suggest an acceptable level of consensus or if CYCLE = MAXCYCLE, then
- The process is terminated with MEW^GM^ being the *Group Means–Ends Network*;
- Otherwise
- Go to Step 6

**Step 6: Analysis and Negotiation**

The procedure above requires methods for estimating group consensus and identifying potential consensus builders, for which we follow the methods presented by Bryson [33]. In addition, the group decision support framework should provision learning for group members who are in a learning mode in the group consensus building process. The framework should also support four essential features required for consensus building: communication, cooperation, information decision guidance, and problem memory. Communications are mediated electronically (e.g., electronic group meetings) in order to ensure the anonymity of group members. In any given cycle, individual preference vectors are private before Step 6, but in the public domain in Step 6. The facilitator can use a priority scheme in order to avoid unwanted broadcasting of positions, and to ensure that high consensus group members have top priority when broadcasting their preferences. For the second feature, cooperation, any exchange of private information requires mutual consent from all relevant parties. A software mechanism releases group members from an agreement to modify preference data if it is dishonored by any of the relevant parties. This involves rolling back the preference data of other members to the values before the agreement. The third feature provides informative decision guidance on individual and group consensus indicators and similarity measure between any pair of preference vectors. Scenario analysis facility is also provided to support the exploration of different scenarios and the generation of mean vectors for any subgroup. The last feature generates problem memory by storing individual preference vectors from each cycle. This enables the user to track the similarity of preference vectors from different cycles, and recall preference vectors from previous cycles.

### 3.4. Identify Analytical Techniques and Goals

Once the group reaches the consensus on the ERM objective, an ontology-based system (described in the next section) can be used to facilitate the identification of analytical goals and associated analytical techniques. While Chen’s approach [38] focuses on knowledge representation, Li et al. [44] designed a Data Mining Model Management (DM^3^) ontology that ontology aims to translate data mining model selection and reuse. More specifically, the DM^3^ ontology provides an ontological representation of analytics goals based on the decision maker’s descriptive statement, which can be utilized in our proposed framework. Each leaf level of MO (as described in Section 3.3) is refined into a set of questions using the GQM approach based on the goal formulation requirements (i.e., object, purpose, focus, viewpoint and context). The questions are presented to the decision maker via the web interface of the application which are mapped to the ontology. An ontology reasoner will then infer the analytical techniques to match the decision problem stated by the decision maker. The results are presented back to the decision maker on the web interface of the ontology-based system.

## 4. Ontology for Identifying Analytical Techniques and Goals

Enterprises constantly struggle to retain and convert tacit knowledge among the workers to organizational knowledge [38]. A knowledge-based system for the representation and storage of tacit knowledge may be beneficial for sharing personal knowledge in an enterprise. Additionally, if the system can utilize reasoning capabilities to generate higher level knowledge, accurate and relevant knowledge can be offered to the knowledge requesters.

An ontology is a formal, explicit specification of a shared conceptualization [45]. It provides a means of explicitly representing domain-specific knowledge in an interoperable format that can be understood by both humans and machines. An ontology-based approach can therefore be used to formally represent KDDA concepts, their attributes and relationships among the concepts. Using ontology as the knowledge model can allow different types of users to share their common understanding and retrieve organizational knowledge for problem-solving and decision support.

Ontology-based decision support for KDDA has several advantages. First, since extensive prior knowledge about the KDDA process and techniques needs to be stored and shared, ontologies provide centralized knowledge presentation and storage (i.e., in a standardized XML/RDF format). They can also be conveniently extended and automatically queried using ontological query language such as SQWRL (Semantic Query-Enhanced Web Rule Language). Hence, ontology-based decision support can provide a common vocabulary in order to unambiguously describe KDDA workflows [46]. Second, as the number of data analytics techniques grows, a collaborative approach wherein individual users can share and upload the background knowledge about KDDA processes is valuable. An ontology can provide such a platform. For example, the DMO (data mining ontology) Foundry [47] constitutes an initial attempt towards a collaborative KDDA knowledge platform. The goal of the DMO Foundry is to integrate and apply various DM ontologies, algorithms and resources that have been developed to support the KDDA process.

An ideal decision support system for KDDA should include an integrated knowledge repository of all relevant prior knowledge. This repository can be implemented as a relational database, or XML databases. XML-based knowledge storage has its advantages as it can support ontological descriptions of operators, meta-data, and workflows, and allows direct querying with XML queries. Current ontologies are implemented in OWL (Ontology Web Language), which supports XML and RDF schema, and greater machine interpretability by providing additional vocabulary along with formal semantics. This interpretability is essential to ensure the extensibility for web-based implementation of KDDA models in a distributed environment [48].

We design an ontology for problem formulation and then describe our ontology-based system for inferencing analytical goals and associated analytical techniques for consensus building. Interested readers can refer to Li et al. [44] for more information on the technical design, implementation and evaluation of the system. The ontology is developed using the Protégé Knowledge Acquisition System [49], a free open source ontology editor and knowledge-based framework developed by the Stanford University School of Medicine. RacePro reasoner plug-in [50] is used for inferencing. The DM^3^ ontology (available at http://webprotege.vcu.edu: 8080/webprotege) is organized in the following manner. The core concepts and relations are developed based on the popular CRISP-DM model, with an emphasis on supporting problem formulation in the BU phase. The ontology is OWL 2 DL (description logic) compliant, allowing decidability and computational inference by reasoner engines such as Pellet and RacerPro. In the rest of this paper, the ontology specific terms are shown in courier new font (e.g., distinct class, inverse object relationship, etc.).

Our comprehensive literature review reveals that current DM ontologies are mainly from a knowledge engineer’s perspective, and mostly capture KDDM-domain specific knowledge, such as data understanding, DM model building and deployment. Our search did not identify any previously published ontology that accurately describes the complexity of modeling the problem formulation in the BU phase. We therefore choose to build the DM^3^ ontology from scratch, while re-using some concepts from previous DM ontologies. We choose the skeletal ontology building methodology proposed by Uschold and Gruniger [51] as it is specific to building ontologies via a manual process. In the light of maturity of ontology design and use, we also incorporate an ontology deployment phase in our design methodology [44].

The main tasks in the ontology design are to determine why the ontology is built, who will use and maintain the ontology, what are the users’ characteristics, what domain will the ontology cover, and what questions will the ontology provide answers for. Within the context of our ontology design, the intended users are environmental risk management users in the organization who may lack sufficient technical knowledge and skills regarding the KDDM processes, techniques, and tools. The DM^3^ ontology provides an ontological representation of DM goals based on the environmental risk management user’s descriptive statements, and helps the identification of analytical goals and associated analytical techniques using its inferencing capabilities.

To capture the knowledge required to describe the analytical goals based on the user’s descriptive environmental risk management objective, the GQM method can be used. GQM requires information about five different components: purpose (motivation behind the goal), focus (quality attribute under study), object (entity under study), viewpoint (entity from whose perspective the goal is designed), and context (scope or environment) [42].

To capture the DM goals in the ontology, we characterize and categorize the first three components of the goal formulation requirement from GQM. Purpose is represented by the DMPurpose class, object is represented by the DMObject class, and focus is represented by the Model Selection Criteria class. Furthermore, the semantic meaning of the descriptive problem statement is inferred from DMPurpose, DMObject and ModelSelectionCriteria, and is characterized by the DMGoal class.

**Figure 2:**
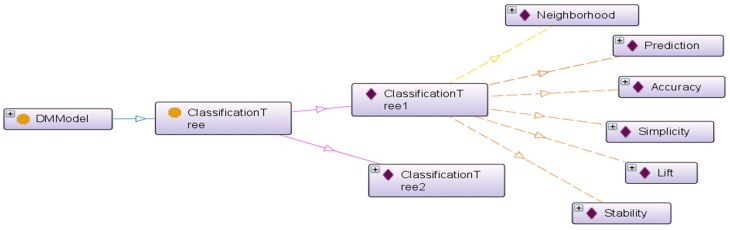
Onto graph representation of DMModel individuals and its object properties.

DMPurpose is related to the DM problem types. Widely accepted classification of DM problem types falls in six categories: classification, estimation, prediction, association rules, clustering, and visualization [52]. DMObject represents the environmental risk management process under investigation, which is similar to fact tables in a data warehousing environment that naturally correspond to environmental risk management process measurement events [53]. Examples of DM objects include customers, products, transactions, etc. Model Selection Criteria are quantified measures used to evaluate the modeling results. The DM^3^ ontology incorporates a shortlist of model selection criteria [26] for different problem types, such as accuracy, simplicity, lift, sensitivity, specificity, etc. Figure 2 shows an Onto Graph representation of an DMModel individual, ClassificationTree1, which has five relevant model selection criteria. This individual is linked with five individuals in the Model Selection Criteria class through its defined object properties. Additional selection criteria can be added to the ontology based on the decision maker’s environmental risk management requirements. Different types of analytical techniques belong to different analytical problem types and have different quantified measures. For example, both regression tree and K-nearest Neighbor (KNN) can be used to provide prediction with interval target variable. However, the regression tree technique provides simplicity measures, while the KNN technique does not. This domain knowledge is built into the DM^3^ ontology and can be utilized to infer applicable analytical goals based on the decision maker’s environmental risk management objectives. 

**Figure 3:**
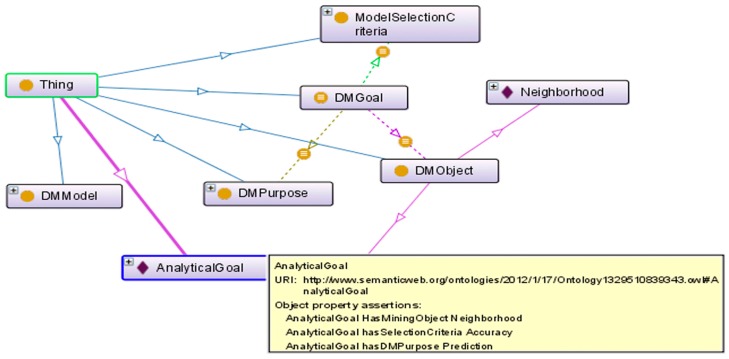
Onto graph Representation of DM^3^ Ontology Inference.

DMGoal class translates the decision user’s DM goals. It captures the semantic meaning of the descriptive problem statement as inferred from DMPurpose, DMObject and ModelSelectionCriteria. Since the viewpoint and the context of the environmental risk management users who use the DM model selection tool is the DM model repository, they are not formally represented in the ontology, as they are the same in the DM goal conceptualization. The ontology will enable the inference of analytical techniques based on described analytical goals, as represented in Figure 3. The inferred relationships are shown in dotted lines, and the defined relationships are shown in solid lines. For example, the AnalyticalGoal has DMPurpose as Prediction, has DMObject as Neighborhood and has ModelSelectionCriteria as Accuracy.

The questions are presented to the urban planner via the web interface of the application which are mapped to the ontology. Responses from the urban planner are mapped to the DMObject, DMPurpose, and ModelSelectionCriteria concepts in the DM^3^ ontology. The ontology reasoner will infer a DMGoal individual if the response satisfies the analytical goal requirement based on the formalism: DMGoal ≡ (has DMPurpose *some* DMPurpose) and (has MiningObject *some* DMObject) and (has SelectionCriteria *some* ModelSelectionCriteria). Once a DMGoal individual is inferred, the ontology reasoner will provide recommendations of the applicable analytical techniques suitable for the analytical goal. The results are presented back to the environmental risk management user on the web interface.

## 5. Case Study

Young et al. [7] demonstrated the value of utilizing social epidemiological methods for analyzing the public’s cumulative exposure and the health effects of HAPs. Specifically, they proposed a model to assess the differential exposure to respiratory, neurological and cancer hazards based on the Townsend Index of socioeconomic deprivation (TSI), regional population size and economic activity, and local population density. To conduct an analysis using the model, data from multiple sources were used, including the National Air Toxics Assessment (NATA) in 2005 [54] (HAPs emissions data), the decennial census data on tract characteristics (data related to county population size, regional population density, concentration of residential population and economic activity), County Business Pattern survey data for all U.S. counties (2005) (for the number employed and annual aggregate wages) and the TSI (for socioeconomic deprivation (SED) measure at the census tract level). The case highlights the issue of complexity in problem formulation, the need for integrating data from multiple sources, and the importance of employing appropriate modeling techniques for KDDA, vis-a-vis, conducting risk assessments of the cumulative health effects of low level chronic exposure to HAPs.

### 5.1. Problem Background Description

Complex and ill-structured business problems affect the results of succeeding phases of the KDDA process. One approach to address this issue is to decompose the complex problem statement into sub-problems to match specific problem solving solution techniques. A problem taxonomy can then be used to categorize the problem statement, problem types, and matching solution techniques. Approaching problem formulation in this manner is particularly useful in the KDDA process which has to account for the plurality of views among multiple stakeholders in a group decision making and negotiation environment.

The U.S. Environmental Protection Agency (EPA) frequently conducts NATA by modeling estimates of the ambient air concentration of subsets of chemical classified as HAPs. They also provide risk estimates of respiratory and neurological health hazard indices (for cancer and non-cancer health endpoints) by quantifying ambient air concentrations of selected HAPs as a function of chemical-specific health effects targeting related organs. While cancer risk estimate models include factors in the toxicological databases for known or suspected carcinogens, substantiated factors are not linked to the non-cancer risk estimate models. The cumulative burden of air pollution exposure raises significant environmental justice policy issues for political leaders, community activists and air quality managers. This pollution hazard also raises major challenges in the field of environmental epidemiology and risk assessments. For example, how to quantify the chemical exposure to populations of lower socioeconomic status? Our understanding of community vulnerability (particularly the confounding of health effects associated with poverty with those related to chemical exposure) may be improved by incorporating issues relating to urban sociology, socio-economic status and social epidemiology into the HAP exposure analysis. Young et al. [54] conceptualized that variation in local neighborhood chemical exposure is decisively influenced by the population size in the region along with the economic activity generated in the region.

### 5.2. Domain Understanding

To conduct ERM in a timely and efficient manner, problem statements have to be first translated into analytical goals. Appropriate analytical techniques applicable to the problem can be identified only after the analytical goals are established. However, an environmental risk assessor may not possess the knowledge in analytics required to translate problem statements into appropriate analytical techniques and algorithms. The assessor may also not have the appropriate technical background to perform the KDDA tasks. Under these circumstances, a system that enables problem formulation by capturing descriptive statements from the ERM decision maker—and helps to identify the analytical goals and associated analytical techniques—would be extremely beneficial.

For assessing the differential exposure to HAPs in the U.S., Young et al. [54] explicitly defined the contexts (i.e., urban counties vs. rural counties), concepts (i.e., level of urbanization, economic activity, degree of SED, and baseline range HAP exposure hazard) and relations among the concepts (i.e., variation in neighborhood chemical exposure and population size, variation in neighborhood chemical exposure and level of regional economic activity, and variation in neighborhood chemical exposure and level of SED). We use the DM^3^ ontology to capture and represent this domain-specific knowledge [44]. For example, in the DM^3^ ontology, conceptualizations such as urbanization, economic activity, SED, and neighborhood are represented as individuals of DMObject in our DM^3^ ontology. Census tract population density, county population size, number employed and aggregate wage are represented as data properties, and relations between concepts are represented as object properties between the individuals.

### 5.3. Model ERM Objectives

In many instances, formulating ERM objectives (FOs and MOs), defining procedures for applying analytical techniques, and evaluating analytical goals are carried out through the process of group consensus. The heuristics for a means–ends network (described in Section 3.3 is useful to elicit group preferences, initiate group discussion on the differential viewpoints, identify measurable attributes for FOs and MOs, to compute consensus indicators, and to terminate the Group MEWs through discourse and negotiation aided by a facilitator. Young et al. [54] identifies a well-defined set of risk management objectives for assessing the differential chemical exposure among vulnerable populations. The FO of the research study was to determine whether SED is confounded with HAP due to their common urban context, or whether SED works to modify HAP exposure. Three MOs are identified to achieve the FO: (1) the determination of variation in the level of HAP respiratory, neurological, and cancer exposure hazard by regional population size and level of economic activity and location; (2) the evidence of differential HAP exposure related to localized SED independent of the regional context of population level and economic activity; and (3), the determination of whether SED is confounded with urbanization and HAP exposure.

### 5.4. Identify Analytical Techniques and Goals

Based on the ERM objectives, the candidate analytical techniques and goals are identified and evaluated. The techniques and goals may then be reviewed by the risk assessor to determine their suitability based on the formulated problem statement. The risk assessor can utilize an ontology based system that uses the DM^3^ ontology for problem formulation. The ontology-based systems can be integrated within an agency’s (e.g., Environmental Protection Agency) intranet for self-service knowledge discovery. Based on the problem formulation needs, the assessor can input a query using the web interface of the ontology based system. The inputs are asserted as individuals (instances of DMObject, DMPurpose, and ModelSelectionCriteria) in the DM^3^ ontology. The reasoner is then triggered to infer the DMGoal individual. The query engine infers all applicable analytical techniques that fit the specific DM goal. The query result is returned to the decision maker via the web interface. To adequately conduct analyses in a timely manner, a thorough understanding of analytical techniques is essential. A system that translates the user’s requirements into analytic technique selection criteria and measures would therefore be useful to enable risk assessors formulate the problem statements and determine the appropriate analytical techniques and goals. Conducting analyses on the approved and supported data repositories would thus be made easier even when the assessor may not possess the appropriate analytical background to perform the knowledge discovery tasks.

## 6. Conclusions

This paper provided a systematic discussion pertaining to problem formulation in the KDDA process and discussed key methods for assessing and supporting decision making and knowledge discovery. An original framework for supporting ERM problem formulation was put forth and an ontology-based approach for identifying analytical techniques and goals was put forth. An environmental epidemiology case study is discussed in order to highlight our proposed framework. By so doing, we addressed several key challenges in providing decision support for problem formulation in the KDDA process. It was discussed that the U.S. Environmental Protection Agency (EPA) estimates hazardous air pollutants (HAPs) and respiratory and neurological health hazard indices (for cancer and non-cancer health endpoints) by quantifying ambient air concentrations of selected HAPs as a function of chemical-specific health effects targeting related organs. It was shown that our understanding of community vulnerability (particularly the confounding of health effects associated with poverty with those related to chemical exposure) may be improved by incorporating issues relating to urban sociology, socio-economic status and social epidemiology into the HAP exposure analysis. However, the cumulative burden of air pollution exposure raises major challenges in environmental epidemiology and the risk assessment of chemical exposure, particularly the confounding health effects associated with poverty with those related to chemical exposure.

In summary, the ontology-based system enables decision makers to formulate analytical problems, describe environmental risk management objectives, and translate them into analytical goals that can be solved by KDDA techniques. Moreover, to addresses limitations in human recall, an ontology-based knowledge system is designed that can provide structured guidance to retrieve relevant knowledge in the problem formulation process, as well as supporting group communication and cooperation, information decision guidance, and problem memory. However, there are a number of challenges associated with data analytics since the quantity of information in the modern era surpasses our harnessing and capture capabilities. Related “Big Data” management challenges involve the storage, mining, sharing, analysis, and display of unstructured or semi-structured data. Accordingly, future work should involve innovative ways to promote the efficient representation, access, and analysis of data. This is particularly urgent in light of the challenges associated with data inconsistency and incompleteness, scalability, timeliness, and security.
